# Large scale single nucleotide polymorphism discovery in unsequenced genomes using second generation high throughput sequencing technology: applied to turkey

**DOI:** 10.1186/1471-2164-10-479

**Published:** 2009-10-16

**Authors:** Hindrik HD Kerstens, Richard PMA Crooijmans, Albertine Veenendaal, Bert W Dibbits, Thomas FC Chin-A-Woeng, Johan T den Dunnen, Martien AM Groenen

**Affiliations:** 1 Animal Breeding and Genomics Center, Wageningen University, Marijkeweg 40, Wageningen, 6709 PG, the Netherlands; 2 Service XS, Plesmanlaan 1d, Leiden, 2333 BZ, the Netherlands; 3 Leiden Genome Technology Center, Human and Clinical Genetics, Leiden University Medical Center, Einthovenweg 20, Leiden, 2333 ZC, the Netherlands

## Abstract

**Background:**

The development of second generation sequencing methods has enabled large scale DNA variation studies at moderate cost. For the high throughput discovery of single nucleotide polymorphisms (SNPs) in species lacking a sequenced reference genome, we set-up an analysis pipeline based on a short read de novo sequence assembler and a program designed to identify variation within short reads. To illustrate the potential of this technique, we present the results obtained with a randomly sheared, enzymatically generated, 2-3 kbp genome fraction of six pooled *Meleagris gallopavo *(turkey) individuals.

**Results:**

A total of 100 million 36 bp reads were generated, representing approximately 5-6% (~62 Mbp) of the turkey genome, with an estimated sequence depth of 58. Reads consisting of bases called with less than 1% error probability were selected and assembled into contigs. Subsequently, high throughput discovery of nucleotide variation was performed using sequences with more than 90% reliability by using the assembled contigs that were 50 bp or longer as the reference sequence. We identified more than 7,500 SNPs with a high probability of representing true nucleotide variation in turkeys. Increasing the reference genome by adding publicly available turkey BAC-end sequences increased the number of SNPs to over 11,000. A comparison with the sequenced chicken genome indicated that the assembled turkey contigs were distributed uniformly across the turkey genome. Genotyping of a representative sample of 340 SNPs resulted in a SNP conversion rate of 95%. The correlation of the minor allele count (MAC) and observed minor allele frequency (MAF) for the validated SNPs was 0.69.

**Conclusion:**

We provide an efficient and cost-effective approach for the identification of thousands of high quality SNPs in species currently lacking a sequenced genome and applied this to turkey. The methodology addresses a random fraction of the genome, resulting in an even distribution of SNPs across the targeted genome.

## Background

The scalability and availability of highly automated genotyping assays for single nucleotide polymorphisms (SNPs) has made the SNP a popular marker in genetic linkage and association studies in a variety of species. In humans, large-scale identification and characterization has resulted in a repository of over 14 million SNPs [1] that are now being used in whole genome association studies to identify genes involved in complex genetic traits [2-6]. The availability of a high quality reference genome sequence and resources to perform low coverage resequencing on a few individuals are prerequisites for the traditional method of whole genome SNP discovery; genomic sequences of different individuals are aligned to a reference genome and nucleotide variation is detected [7]. Although very effective in species whose genome has been sequenced, such as human, cow, horse, and chicken, for the majority of species this method of SNP discovery is currently not feasible. Although second generation sequencing has lowered the cost per sequenced base a hundred-fold and allows the resequencing of complete genomes in a fraction of the time, the size of the sequencing target still exceeds the frequently available budget. By deep sequencing reduced representation libraries (RRL), SNPs can be discovered and allele frequencies estimated more economically [8]. The complexity of a pool of DNA samples from multiple individuals is reduced by two orders of magnitude [9] by isolating a fragment size range of a complete endonuclease digestion. Depending on the applied endonuclease, the obtained RRL contains hundreds of thousands of fragments within the optimum size range of the sequencing platform, equally distributed over the genome and with a low representation of repetitive elements. Tens of thousands of high quality SNPs can be identified by aligning the sequence reads that result from deep sequencing the RRL to a genome reference sequence. This approach already has been applied to species with a more or less completed genome draft sequence, like cow [8], as well as on species in which genome sequencing is ongoing, such as pig [10].

However, many species, such as turkey, are still lacking a completely sequenced genome. Although high-throughput sequencing technologies are rapidly evolving and have drastically lowered the cost of whole genome DNA sequencing, the de novo assembly of a mammalian-sized genome remains a challenge [11]. Despite the number of published algorithms for short fragment de novo sequence assembly [12-16], which assembles whole prokaryotic genomes [17,18], reconstructing the sequencing targets of hundreds of megabases will require parallelization of these algorithms. Furthermore, many of these species still lack sufficient genetic markers and linkage maps that would aid in the ordering of the sequencing contigs and anchoring the contigs to specific chromosomes. Thus, the development of an efficient method for SNP discovery in such species is of high importance. We provide an effective strategy for combining RRL deep sequencing with de novo contig assembly based on next-generation sequencing data. The key of our approach is based on using RRLs consisting of large fragments (2-3 kbp) and random shearing. Performing high-throughput sequencing to a sufficient depth on sheared RRL in a pooled DNA sample in the first place enables reconstruction of the sampled genome fraction by de novo sequence assembly. The assembled contigs subsequently serve as a reference genome to which all short reads derived from multiple individuals can be mapped accurately, and SNPs can be called reliably [19].

The aim of this study was to develop an extremely cost effective method to detect high quality SNPs in unsequenced genomes. We applied this method to turkey, a species of considerable economic importance, and used the genome of a closely related species, chicken [20-22], to benchmark our approach.

## Results

### RRL preparation

We prepared a pooled DNA sample consisting of DNA samples from six turkey individuals. A RRL was prepared by digesting the pooled DNA sample with *Sau*3A and isolating the fragments in the size range of 2-3 kbp. This fraction consists of an estimated 5-6% of the turkey genome. The turkey genome has a high similarity to the chicken genome and is approximately the same size (~1.2 Gbp). Therefore, the isolated 5-6% fraction of the turkey genome represents approximately 62 Mbp. This estimate was confirmed by selecting all 2-3 kb fragments of an in silico *Sau*3A digest of the chicken genome build WASHUC2, which resulted in a total of 27,025 fragments representing 63.4 million bases. The turkey RRL was sequenced using the Illumina sequencing technique [23] after random shearing of the isolated *Sau*3A fragments. The resulting data set of short sequence reads forms the basis for contig assembly, providing sufficient sequence context flanking the SNPs to allow for the subsequent development of SNP genotyping assays.

### DNA sequencing and sequence filtering

We generated 114 million sequence reads of turkey genomic DNA using the Illumina Genome Analyzer. The resulting 36 bp sequence reads were trimmed to 32 bp because of the decay in base-call quality observed after the 32^nd ^base. Subsequent removal of sequence reads with non-called bases resulted in almost 108 million reads, providing an estimated 56-fold coverage of 5-6% of the turkey genome. We used *Sau*3A to generate the RRL and, as expected, we observed that a fraction of the sheared DNA fragments started with the GATC restriction tag (Table 1), though the observed frequency was higher than expected. We discarded 984,258 reads tagged as repeat by RepeatMasker [24]. Reads that were, based on the theoretical coverage, over-represented more than four times were also removed because of their likeliness to resemble repetitive sequences or to represent duplicated regions in the turkey genome. Besides not being able to properly reconstruct repeats without mate-pair information at this low genome coverage, we also wanted to avoid false SNP predictions due to paralogous sequences. To improve the accuracy of the turkey genome assembly and reliably predict SNPs on the assembled contigs, data were screened for quality by applying a maximum sequencing error tolerance for reads with a single representation. For assembly purposes, we only tolerated one sequencing error per 100 bases, whereas one error per ten bases was tolerated in the reads used for SNP detection. After removing repetitive, overabundant, and low quality sequences with a single representation, almost 27 million reads (864 million bp) corresponding to 8.6 million unique sequences remained for contig assembly. For SNP detection purposes, almost 33 million reads (1.05 billion bp) corresponding to 13.8 million unique sequences passed our thresholds.

**Table 1:** Summary of DNA sequence filtering results.

	**Filter applied**^1^	**Pass Filter**	**(%)**	**GATC start (%)**
**Pre-selection**	l32 n.	107888201	94.48	24.78
**Assembly**	l32 n. q20 o230	27979963	24.50	46.64
**SNP**	l32 n. q10 o230	32941906	28.84	40.23

^1 ^sequences are filtered for length 32, without base-call errors (n or .). Singly represented reads are required to have a per base-call quality score of 20 (assembly data set) or 10 (SNP data set). Sequences more than four times overrepresented, based on the expected 56× coverage, were discarded.

### Reference genome construction

**Figure 1:**
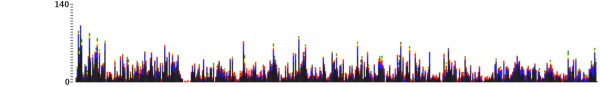
**Distribution of short read turkey contigs, turkey BES, and SNPs on chicken chromosome 4**. In black, short read contigs <100 bp; in blue, short read contigs ≥ 100 bp; in red, BES; in yellow, BES-short-read contigs; and in green, SNPs. On the X-axis, the chicken genome in 200 **k**b intervals. On the Y-axis, the frequency of mapped turkey features for a specific chicken genome interval.

For the actual SNP detection, a required reference genome was constructed by first performing de novo short read sequence assembly. Available de novo assemblers were SSAKE [12], SHARCGS [13], Edena [14], Velvet [15], and ALLPATHS [16]. Likely because of the large genome target and relatively high error rate of 1% ALLPATHS and SHARCGS showed an unfeasible large memory footprint and runtime. Probably because of the relatively low genome target coverage (14×) Velvet did return only 24 assembled sequence contigs all of which had a more than 15× coverage. Although Edena assembled contigs computationally more efficiently, SSAKE resulted in a higher number of assembled sequences and longer sequence contigs (Table 2). Based on these results, the final assembly of the reference genome was performed with SSAKE. Using SSAKE [12], we assembled 36,163,074 bp into 627,600 short sequence contigs, with an average coverage of 9.52 and an N50 of 53 bp using the default assembly parameters. The quality of the reference genome assembly was estimated by mapping the short sequence contigs of at least 50 bp (further referred to as c50) to the draft genome sequence of chicken, the most closely related species [20-22] for which a genome sequence is available. As a benchmark, we used 20,000 publicly available turkey BAC-end sequences (BES) (Table 3). Direct alignment of the 35 bp Illumina reads resulted in the unique alignment of approximately one-third of the sequences. This fraction of turkey sequences uniquely aligning to the chicken genome steadily increased with increasing contig length, until reaching a maximum of 73% for contigs in the size range of 100-150 bp. At contig lengths above 150, this percentage gradually decreases dropping below 10% for contigs of 1000 bp and larger. The short sequence contigs and BES within the size range of 100-300 bp had comparable mapping statistics. The observed trend of a decrease in alignment for the larger assembled contigs was not observed for the BES. The distribution of the assembled contigs across the turkey genome was evaluated by aligning the contigs against the chicken genome. The contigs were distributed uniformly across the genome (Figure 1 and Additional file 1). The mapping results were subsequently used to further improve the assembly by merging contigs that mapped to adjacent or overlapping locations on the chicken genome. Merging of these contigs resulted in a more contiguous reference sequence and an increase in the average size of the assembled contig (this assembly is further referred to as c50ca). We detected 15,754 adjacent or overlapping contigs, 13,695 identical overlaps, and 24,593 contigs in total were merged into 10,898 bigger contigs, representing 2,072,380 nucleotides and a N50 of 198 bp. Finally, we further extended our turkey reference genome (referred to as c50caB) by including the publicly available BES. A total of 5,831 BES (2,840,087 bp) with 49,638 short sequence contigs (4,032,887 bp) assembled into 8,526 new contigs with a total sequence length of 3,022,857 base pairs. The remaining 38,957,511 bp of the genome sequence was represented by 578,885 singletons. The BES, as well as all contigs from the extended assembly, were aligned to the chicken genome sequence by using BlastZ [25] to predict their distribution within the turkey genome (Figure 1 and Additional file 1).

**Table 2:** Short read assembly results.

	**algorithm^1 ^and non-default parameters**		
	edena -c 33 -m 16	velvet 15^2^	SSAKE
**contigs**	230741	24	627600
**assembled reads**	8965681	NA	13964267
**assembly length**	17487533	2812	36163074
**N50**	90	129	53

^1^algorithm versions were: edena-2.1.1, velvet_0.3, SSAKE_v2.0

^2^parameter applies to hash_length

**Table 3:** Quality estimation of turkey short read contigs based on alignment to the chicken genome.

	**frequency**^1^		**percentage mapping to chicken**^2^							
		
**size**	**contigs**	***BES***	**uniquely**		**with secondary hit**		**multiple hits**		**total**	
**50-70**	124480	*0*	47	-	2	-	1	-	50	-
**75-100**	38382	*1*	53	*0*	2	*0*	1	*0*	56	*0*
**100-150**	25808	*156*	73	*69*	1	*4*	0	*4*	74	*77*
**150-200**	8878	*226*	69	*78*	1	*6*	0	*4*	70	*88*
**200-300**	6453	*835*	63	*82*	1	*3*	0	*3*	64	*88*
**300-400**	2372	*2428*	51	*86*	1	*4*	0	*1*	52	*90*
**400-500**	1192	*6664*	40	*87*	1	*3*	0	*2*	40	*92*
**500-600**	682	*8509*	30	*88*	1	*3*	0	*2*	31	*93*
**600-800**	688	*1510*	18	*88*	0	*4*	0	*2*	18	*93*
**800-1000**	308	*54*	11	*83*	0	*0*	0	*2*	11	*85*
**>= 1000**	380	*4*	6	*80*	1	*0*	0	*20*	7	*100*

^1 ^frequency in which contigs and BES (in italics) occurred per size category

^2 ^per size category percentage of contigs and BES (in italics) that mapped to the chicken genome

### SNP discovery

**Figure 2:**
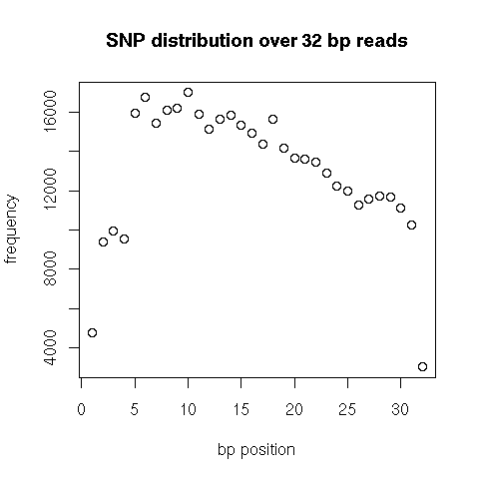
**Distribution of nucleotide polymorphisms across 32 bp genome analyzer reads**. The X-axis represents the 32 base sequence read. On the Y-axis **is **the cumulative number of identified SNPs per base position of the sequence read.

**Figure 3:**
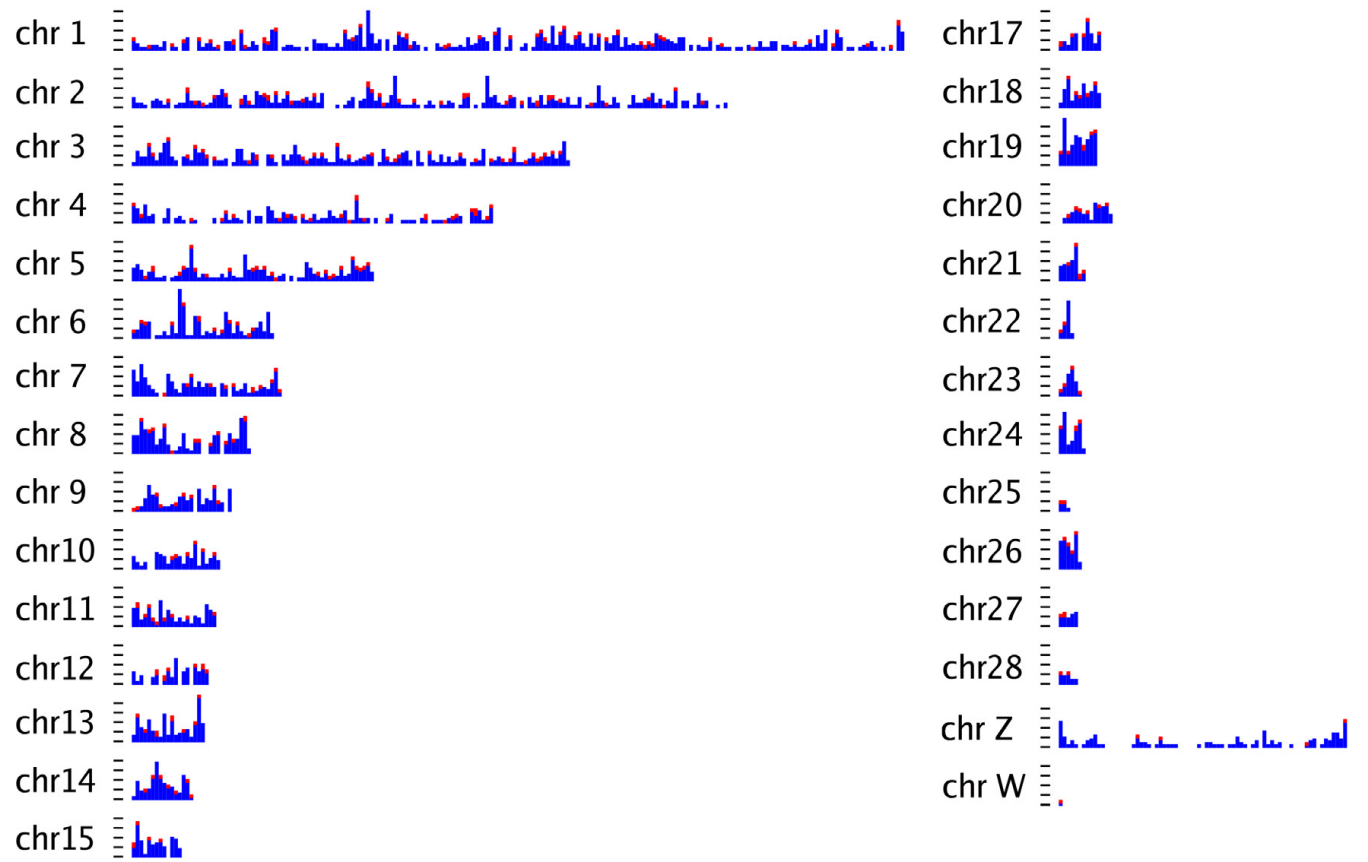
**Distribution of 6,134 SNPs that mapped uniquely to the genome, 343 of which were selected for validation**. In blue, 5,791 putative SNPs identified using the c50caB reference **sequence **and mapping uniquely to the chicken genome; in red, 343 uniquely mapping putative SNPs selected for validation. On the X-axis, the chicken genome in 1 Mb intervals. On the Y-axis, the frequency of mapped putative turkey SNPs for a specific chicken genome interval.

We aligned 32,941,906 reads (Table 1) to each of the three reference genomes described above (c50, 50ca, and c50caB). We adjusted the alignment parameters towards an approximately uniform distribution of nucleotide variation over the 32 bp reads using reference c50 (Figure 2). Putative SNPs within sequence clusters with a sequence depth less than four times the maximum theoretical coverage (58×), and in which the minor allele was represented at least three times, were recorded. Using these parameters, we identified 7,617 SNPs residing in 6,696 contigs out of the 209,623 contigs of the c50 reference. By using the C50ca assembly, 321 additional SNPs were detected (Table 4); furthermore, the fraction of SNPs with a sufficient flanking sequence increased considerably. Finally a further increase in the number of SNPs was achieved by using the reference assembly that included the BES (c50caB). This reference consisted of 192,731 contigs of which 7,952 contained one or more SNPs. Putative SNPs detected in uniquely mapped reference sequence contigs were plotted along the chicken chromosomes. Alignment with the chicken genome showed that the identified putative SNPs were distributed uniformly across the genome (Figure 3).

**Table 4:** Overview of SNPs identified.

			**nt flanking sequence**^1^		
**reference**^2^	**Contigs**	**SNPs**	**40/40**	**20/20**	**2/40 **
**c50**	209623	7609	2254	5218	6454
**c50ca**	195928	7930	2760	5636	6834
**c50caB**	192731	11287	5620	8902	10125

^1^40/40 refers to SNPs that are flanked on both sides by at least 40 nucleotides of genomic sequence.

20/20 refers to SNPs that are flanked on both sides by at least 20 nucleotides of genomic sequence.

2/40 refers to SNPs that have at least 2 nucleotides flanking sequence on one side and at least 40 nucleotides on the other.

^2^c50 refers to reference genome consisting of short read contigs of 50 bp or more

c50ca is extended genome assembly based on chicken alignment

c50caB is extended genome assembly based on chicken alignment and turkey BES.

### Validation

The application of the chicken reference genome in the improvement of our turkey reference, in which turkey contigs were merged based on comparative alignment results, requires conservation between these two genomes. Chicken and turkey genome conservation was determined by performing PCR amplification with forward and reverse primers designed on 13 neighboring short read turkey contigs aligning up to 0.5 kb apart on the chicken genome. As a control, PCR was performed on the corresponding chicken DNA target for which additional primer pairs were developed in the case that the turkey primers were not cross species applicable (Additional file 2). The resulting PCR products on the turkey genome were compared with corresponding amplification products obtained on the chicken genome; they were approximately the expected length based on the chicken genome (Additional file 2).

The contig assembly and SNP detection procedure were initially validated by PCR amplification and subsequent sequencing of the fragments in the six turkey individuals that made up the DNA pool from which the short read sequence data set was generated. Primers were developed on 12 contigs, each containing multiple putative SNPs. All 29 SNPs predicted on these 12 contigs were confirmed. In addition, a further five additional SNPs were identified (Additional file 2).

Further SNP validation was done by genotyping an animal panel consisting of 96 animals using 343 predicted SNPs distributed uniformly over the chicken genome (Figure 3) and 41 randomly selected SNPs that did not map uniquely to a single location in the chicken genome. A total of 340 SNPs gave reliable genotypes in the assay, and 96% of these were polymorphic (Table 5). We observed that SNPs predicted within contigs that uniquely mapped to the chicken genome had a more than five times higher chance of giving reliable genotypes than SNPs from contigs that aligned to multiple locations in the genome. The minor allele count (MAC) of each polymorphic SNP, the minor allele frequency (MAF) observed in the six animals represented in the discovery pool, and the MAF based on all 96 genotyped individuals are shown in Additional file 3. The average MAF of all successfully typed SNPs was 0.28, and the average heterozygosity in the individuals typed was 0.35. The correlation between MAC and MAF was 0.69 in the six animals that made up the discovery pool.

**Table 5:** SNP performance statistics.

**SNP performance**	**384 selected SNPs**			
	**Mapped (343)**	**%**	**Unmapped (41)**	**%**
**polymorphic**	304	88.6	20	48.8
**monomorphic**	12	3.5	4	9.8
**not working**	27	7.9	17	41.5

Genotyping performance of 343 SNP discovered in short read contigs that were uniquely mapped on the chicken genome and 41 SNPs discovered in contigs that were not, or not uniquely, mapped on the chicken genome.

## Discussion

### Next generation sequencing

Our large-scale nucleotide variation study on the turkey genome, including a partial assembly of a reference genome, demonstrates that short fragment second-generation sequencing of randomly sheared large fragment RRLs is an efficient and cost-effective approach for SNP discovery, providing thousands of high quality SNPs, even in the absence of an available genome sequence. This approach combines the advantages of using an extremely cost-effective sequencing platform with the ability to provide SNP sequence context by short fragment assembly. The sequence context provided by this SNP detection approach makes this the ideal method for the development of SNP assays on a variety of genotyping platforms for all species without sequenced genomes.

We had to discard nearly 75% of our sequence data to meet quality constraints for the sequence assembly (Table 1). This was in pair due to suboptimal sequence densities resulting in suboptimal clustering on the tiles of the Illumina Genome Analyzer (see methods section), resulting in poor sequence quality and low sequence output. On top of this, a relatively large proportion (about half of the sequences passing our quality thresholds) started with the endonuclease cleavage site. The underestimation of this fraction in the initial length trimmed sequencing data subset was most likely caused by sequencing errors in the first four bases of a read. Stringent filtering of reads revealed the real ratio and provided a higher quality data subset, but lowered the total theoretical coverage of our sequencing target to 10×. To avoid this observed bias towards the ends of the RRL fragments, an option is to dephosphorylate the ends of the RRL restriction fragments prior to random shearing and ligation to the sequencing adaptors. We were only able to assemble roughly 60% of our sequencing target covered by our RRLs, most likely due to the limited sequence depth (10×) of our final data set after using stringent quality thresholds. The recent addition of paired end sequencing to second generation sequencing, the increased read length and the predicted further increase in sequence length and tens of gigabases of useful sequence data per machine run in the near future [23,26], will allow more efficient sequence assembly. This will result in increased coverage of the sequence target and an increased contig length of the assembled sequences, at lower costs. An improved assembly allows a substantial increase in the number of SNPs identified, as well as a considerable increase in the number of SNPs for which a genotyping assay can be designed. Another strategy to increase the number of assayable SNPs would be to use combination of two different sequencing platforms, such as Roche 454 and Illumina GA, in which longer reads (454) are being used for reference construction and short reads provide the necessary sequencing depth to detect nucleotide variation.

### Benchmarking and improving

We showed that the genome sequence of a closely related species can be used for benchmarking the assembled contigs, the genome coverage and can further improve the reference assembly by merging contigs mapping to an adjacent location on the genome of that particular species. In the case of turkey, we applied this cost-effective strategy by using the likewise Galliform genome of the chicken. Previous studies indicate that chicken and turkey karyotypes (common ancestor ~28 MYA) have undergone relatively very few chromosomal rearrangements during evolution compared to mammals [20]. Moreover, results of cross species hybridisation studies and comparative genomics suggest that chicken an turkey share a high sequence identity [20-22] which makes the chicken genome sequence usable to benchmark the turkey reference assembly.

An assessment of the quality of our assembled turkey contigs was done by mapping the contigs to the chicken genome and comparing the results with the alignment statistics of turkey BES of the same size range. The results indicate that the contigs up to 300 bp, in general, are of good quality and that turkey BES share high sequence identity with the chicken genome. The comparison between the assembled contigs and BES indicate that most of these contigs represent valid sequences of the turkey genome. At increasing contig length, the number of sequences that align uninterrupted to a unique location in the chicken genome declines, dropping below 10% for contigs in the size range of 1000 bp. The fact that this decline is not observed for the turkey BES indicates that it is not due to small indels between the chicken and turkey sequences, but that this is an artifact caused by the assembly. These results indicate that at increasing contig lengths, the chance of mis-assemblage by SSAKE increases exponentially. However, because most SNP typing assays make use of the sequences directly flanking the SNP, this will only have a small effect on the success rate of the genotyping assays. At total of 7609 SNPs were identified on the assembled short read contigs of which 84% was flanked by sufficient sequence to allow probe design in a genotyping assay. To make the turkey reference more contiguous we used the chicken genome to identify contig pairs that uniquely mapped adjacent to each other, showing a small overlap. In 87% of these cases, overlapping contigs appeared to have identical sequences within the overlapping region. Although biased by the alignment algorithms used, which remove unaligned tailing ends of contigs, our comparative assembly results suggest that the mapped contigs are of a constant high quality and can be mapped with high accuracy. Therefore, these results allow the merging of the smaller contigs, resulting in a significant increase in the average length of the assembled turkey contigs. The resulting reference sequence appeared to be beneficial in the identification of SNPs and, in particular, increased the number of SNPs with sufficient flanking sequence for designing a genotyping assay. This benefit is clearly illustrated by the 4% increase in the total number of SNPs identified and 22% increase in SNPs with at least 40 bp of flanking sequence on both sides. The alignment of the turkey contigs with the highly similar chicken genome also turned out to be a good predictor of genotyping success rates for the SNPs (Table 5). The SNPs located on turkey contigs that aligned to more than a single location on the chicken genome appeared more likely to fail in the genotyping assay than SNPs located on uniquely aligning turkey contigs which is probably because these are likely to contain duplicated sequence or repetitive sequences of the turkey genome. Repetitiveness of turkey and chicken genome sequences were compared by applying the IR [27] algorithm on the available turkey BES (9,9 Mb) and 20,000 (8,3 Mb) chicken genomic sequences randomly selected from the NCBI database (data not shown). Obtained non-normalised *I*_r _values suggest that the turkey genome is slightly less repetitive (0.6247) than the chicken genome (0.7126). The average *I*_r _for the chicken genome was 0.3905 and ranged from 0.0793 in chromosome 19 to 1.3419 in chromosome 16. Compared to other eukaryotes like Human, Mouse and Arabidopsis [27] the chicken genome is at least three times less repetitive which is in line with the results of a previous study in which repeats were computationally identified on the chicken genome [28]. This lower level of repetitiveness is beneficial for the genotyping success rate because of the lower occurrence of false SNP predictions due to repetitive genomic regions.

To further maximize the number of identified SNPs, the available turkey BES were added to the reference genome. Again, these additional sequences not only resulted in the identification of an additional 3357 additional SNPs, they also increased the number of SNPs with a sufficient amount of flanking sequence. The assembly of short read contigs and BES resulted in, at least, a 25% reduction of sequence redundancy in the assembled short read contigs. Removal of sequence redundancy in the reference genome is beneficial for downstream SNP detection because of the reduction in the number of sequence reads being assigned ambiguously to multiple locations on the reference genome during the alignment. SNPs predicted within sequence clusters containing these ambiguously mapped reads are indistinguishable from falsely predicted SNPs due to the clustering of paralogous sequences and thus discarded.

### Allele frequencies

Our conservative approach requiring a minimal MAC of three was designed to minimize false positive SNP discovery and, consequently, ignored large numbers of less abundant true nucleotide variations. The five additional SNPs we identified by PCR and sequencing that were not previously detected in silico are a typical consequence of applying a minimum redundancy cut-off. However, the selection for SNPs with a MAC of at least three drastically reduces the chance that sequencing errors are considered an SNP. Keeping the number of false positives as low as possible in general is more important than maximizing the number of SNPs. True nucleotide variation might also be lost during sequence assembly in which contigs are extended by a read only if the consensus base ratio is 0.6 or more. Single nucleotide polymorphisms with a MAF higher than 0.4 very likely break the contig extension; for this reason, they will only be detected on the tailing ends of assembled contigs. The absence of sequence context on one side of these polymorphisms further hampers the alignment of additional reads to form deep sequence clusters meeting the minimum allele count constraint applied during SNP detection. This concept explains the increase in the number of SNPs discovered on the extended reference genome though the number and total number of base pairs covered decreased. The occurrence of a few SNPs with an estimated MAC higher than 0.4 can be explained by a lower MAC in the assembly data subset compared to the MAC in the SNP detection data subset.

## Conclusion

Our strategy of assembling a reference genome from short next-generation sequences of a randomly sheared RRL of pooled genomes, followed by subsequent SNP detection by aligning the same short reads against this reference genome, is a cost-effective and efficient method for the high rate discovery of SNPs in species with unsequenced genomes. The availability of a closely related sequenced genome is not a requirement but comparative mapping facilitates the selection for high quality SNPs. Our comparison with the chicken genome further suggests that the high quality SNPs identified in this report most likely cover the complete turkey genome and provide the first large SNP resource for genetic studies in turkey.

## Methods

### Library construction

Genomic DNA was extracted from the blood of six unrelated F_0 _individuals from a male and a female turkey line, selected for growth and reproduction characteristics respectively, three samples from each line. The selection of the restriction enzyme was based on the 10 to 20-fold reduction of genome complexity in the 2-3 kb size region run on a 1.5% agarose gel. Ten enzymes were tested (*Sau3A, XhoI, AvaI, MspI, SacI, KpnI, SalI, AluI, TagI*; New England Biolabs, Ipswich, MA, USA); of which, *Sau3A *was finally selected to make the Turkey RRL because of good digestion performance and a desired 5-6% fraction of the genome in the 2-3 KB size range. In total, 100 μg of the pooled DNA was digested using 1,000 units of the restriction enzyme *Sau3*A in a total volume of 240 μl. The digested pooled DNA sample was fractionated on 1.5% low melting point agarose gel at 100 V for 3 hours and stained with ethidium bromide. The 2-3 kb sized fraction was sliced out of the gel, melted, and loaded on a new 1.5% low melting agarose gel for another fractionation at 100 V for 1 hour. The 2-3 kb fraction was sliced out of the gel and the DNA was recovered by β-Agarase-I treatment, purified by phenol-chloroform extraction, and precipitated with 2-propanol. DNA was dissolved in TE with a concentration of 50 ng/μl. The isolated DNA was randomly sheared, end-repaired, and prepared using the Illumina Sample preparation kit [29].

### Sequencing

Five picomole aliquots of the library were processed with the Illumina Cluster Generation Station (Illumina Inc., USA) following the manufacturer's recommendations. The Illumina IG Genome analyzer (Illumina Inc., USA) was programmed to produce a theoretical fixed read length of 36 bp. Images were collected over 4,040 tiles, each of which contained 685-41,954 clusters.

### Sequence filtering and reference assembly

Reads were trimmed to 32 bp, and reads with an occurrence of more than four times the theoretical coverage were discarded. Two data sets were created; one was the assembly data set and the other the SNP detection data set. In the SNP detection data set, we required a per base quality score of at least 10 if the read was singly represented. For the assembly data set, we required that a particular 32 bp sequence be represented two times or that every base in the 32 bp sequence have a quality score of at least 20.

Furthermore, the assembly data set was analyzed for repetitive elements using RepeatMasker [24] with default options, species chicken, and reads containing repetitive elements were removed. Remaining reads were assembled to short read contigs using SSAKE [12] and the default parameters. The data set containing contigs larger than 50 bp are referred to reference genome c50.

The short read contigs (c50) were mapped on the chicken genome with the selection criteria that a contig had to align along 80% of its length with at least 60% identity. Short read contigs in the size range of 50-100 bp were mapped using Megablast [30], and short read contigs of 100 bp and longer were mapped using BlastZ [25]. Mapping results were parsed using a custom made Perl script to identify short read contigs that mapped adjacent or with a less than 21 bp identical overlap. These identified contigs were subsequently merged, and this data set is referred to as reference genome c50ca.

The turkey genome reference sequence was further extended by adding 20,388 publicly available BES of the CHORI-260 turkey BAC library [31] to all short read contigs (data set c50ca) and assembled using phrap [32] and the default parameters. Obtained sequences larger than 50 bp were used as a turkey reference genome in the SNP detection procedure and referred to as c50caB.

### SNP detection

The SNP detection was performed with MAQ [19] (default parameters) using the SNP detection data set and one of the reference genomes (c50, c50ca, or c50caB). Putative SNPs were tagged if the reads involved were mapped unambiguously on the reference genome and the minor allele appeared at least three times. The SNPs were discarded if the depth exceeded four times the theoretical sequence depth, the consensus quality of the SNP was less than 30, or the best mapping read in the sequence cluster had a mapping score lower than 60.

### Validation

Validation of the assembled contigs and detected SNPs was performed two ways.

First, PCR primers were designed for 12 contigs containing multiple SNPs using primer 3. The PCR was performed in 12 μl and contained 6 μl Abgene 2× PCR Mastermix (ThermoScientific), 60 ng template DNA, and 4 pmol of each of the two primers. The PCR cycling conditions were 95°C for 5 min, 35 cycles of 30 s at 95°C, 45 s at 55°C, and 90 s at 72°C, followed by a final elongation step of 72°C for 2 min.

The PCR products of the six animals from the discovery panel were purified using millipore PCR cleanup filter plates (MSNU03050) and sequenced using the DETT sequencing kit according to the manufacturer's specifications (GE Healthcare).

Unincorporated dye terminator was removed by ethanol precipitation and analyzed on a 48-capillary ABI 3730 DNA analyzer (Applied Biosystems). Sequencing results were further analyzed with the STADEN Package.

The second method of validation was genotyping the SNPs using the Illumina GoldenGate^® ^Genotyping assay on an Illumina^® ^BeadXpress with veraCode™ technology. Selection criteria for the SNPs were based on the Illumine design score (above 0.8) and MAC ranging from .5 to .15 detected by MAQ [19]. For the total 384 SNPs assayed, including 343 SNPs equally distributed along the chicken genome and 41 randomly selected SNPs that did not map to a single location in the chicken genome, oligonucleotides were designed, synthesized, and assembled into oligo pooled assays (OPA) by Illumina Inc. The 384 SNPs were genotyped in 96 animals which included the six F_0 _animals from the discovery panel and 29 additional F_0 _animals and further consisted of 47 F_1 _animals and 14 unrelated animals derived from 2 inbred lines. Genotyping results were analyzed in Beadstudio.

The correlation between allele frequency estimated by sequencing and genotyping was calculated over 310 observations (Additional file 3) by randomly selecting the major or minor allele.

## Availability and requirements

The SNPs identified in this study, in which the polymorphism is flanked by 20 bp of sequence context on each side, have been deposited in the National Center of Biotechnology (NCBI) SNP database (dbSNP) under submitter handle WU_ABGC. NCBI_ss 142460378-142468928 excluding (142463311, 142463314, 142463316, 142463318, 142463320, 142463322, 142463324, 142463326, 142463328, 142463330, 142463332, 142466905, 142466907, 142466910, 142466912) represents predicted SNPs that were not tested in our animal panel. Predicted SNPs that were confirmed are listed in Additional file 3. The SNPs with less than a 20 bp sequence context will be available upon request.

## Authors' contributions

HHDK designed and developed the genome reference construction method and SNP prediction method and wrote the manuscript. AV, BWD, and RPMAC collected and prepared the samples and performed the initial validation and genotyping analysis. TFCC, JHD, and RPMAC coordinated and supervised the DNA sequencing. MAMG and RPMAC coordinated and supervised the experiment implementation and assisted in the manuscript preparation. All authors read and approved the final manuscript.

## Supplementary Material

Additional file 1 **Distribution of short read turkey contigs, turkey BES, and SNPs on chicken chromosomes**. In black, short read contigs <100 bp; in blue, short read contigs ≥ 100 bp; in red, BES; in yellow, BES-short-read contigs; and in green, SNPs. On the X-axis, the chicken genome in 200 kb intervals. On the Y-axis, the frequency of mapped turkey features for a specific chicken genome interval. Click here for file

Additional file 2 **Primer sequences, PCR product sizes, and number of SNPs confirmed per amplicon for the 25 loci evaluated for genome similarity and SNPs**. ^1^C = chicken, T = turkey. First, 13 loci were used in the determination of genome conservation between chicken and turkey. Next, 12 loci were used to validate the contig assembly and SNP detection procedure. Click here for file

Additional file 3 **Validation of candidate SNPs by genotyping a panel consisting of 96 turkeys**. The 96 animals included the six turkeys from which the pooled DNA sample for SNP discovery was prepared. Listed are the 324 polymorphic SNPs out of 340 predicted SNPs that resulted in a working assay. For all SNPs, the minor allele frequencies (MAF) were calculated for the group of six turkeys from which the pooled DNA sample was prepared and for the total panel. Also, the fraction of individuals heterozygous for an SNP locus was calculated. Click here for file

## References

[B1] Sherry ST, Ward M, Sirotkin K (1999). dbSNP-database for single nucleotide polymorphisms and other classes of minor genetic variation. Genome Res.

[B2] Trikka D, Fang Z, Renwick A, Jones SH, Chakraborty R, Kimmel M, Nelson DL (2002). Complex SNP-based haplotypes in three human helicases: implications for cancer association studies. Genome Res.

[B3] Sawcer S, Ban M, Maranian M, Yeo TW, Compston A, Kirby A, Daly MJ, Jager PLD, Walsh E, Lander ES, Rioux JD, Hafler DA, Ivinson A, Rimmler J, Gregory SG, Schmidt S, Pericak-Vance MA, Akesson E, Hillert J, Datta P, Oturai A, Ryder LP, Harbo HF, Spurkland A, Myhr K, Laaksonen M, Booth D, Heard R, Stewart G, Lincoln R, Barcellos LF, Hauser SL, Oksenberg JR, Kenealy SJ, Haines JL, Consortium IMSG (2005). A high-density screen for linkage in multiple sclerosis. Am J Hum Genet.

[B4] Burton PR, Clayton DG, Cardon LR, Craddock N, Deloukas P, Duncanson A, Kwiatkowski DP, McCarthy MI, Consortium WTCC, (TASC) ASC (2007). Association scan of 14,500 nonsynonymous SNPs in four diseases identifies autoimmunity variants. Nat Genet.

[B5] Meyre D, Delplanque J, Chèvre J, Lecoeur C, Lobbens S, Gallina S, Durand E, Vatin V, Degraeve F, Proença C, Gaget S, Körner A, Kovacs P, Kiess W, Tichet J, Marre M, Hartikainen A, Horber F, Potoczna N, Hercberg S, Levy-Marchal C, Pattou F, Heude B, Tauber M, McCarthy MI, Blakemore AIF, Montpetit A, Polychronakos C, Weill J, Coin LJM, Asher J, Elliott P, Järvelin M, Visvikis-Siest S, Balkau B, Sladek R, Balding D, Walley A, Dina C, Froguel P (2009). Genome-wide association study for early-onset and morbid adult obesity identifies three new risk loci in European populations. Nat Genet.

[B6] Rafnar T, Sulem P, Stacey SN, Geller F, Gudmundsson J, Sigurdsson A, Jakobsdottir M, Helgadottir H, Thorlacius S, Aben KKH (2009). Sequence variants at the TERT-CLPTM1L locus associate with many cancer types. Nat Genet.

[B7] Li G, Ma L, Song C, Yang Z, Wang X, Huang H, Li Y, Li R, Zhang X, Yang H, Wang J, Wang J (2009). The YH database: the first Asian diploid genome database. Nucleic Acids Res.

[B8] van Tassell CPV, Smith TPL, Matukumalli LK, Taylor JF, Schnabel RD, Lawley CT, Haudenschild CD, Moore SS, Warren WC, Sonstegard TS (2008). SNP discovery and allele frequency estimation by deep sequencing of reduced representation libraries. Nat Methods.

[B9] Altshuler D, Pollara VJ, Cowles CR, Etten WJV, Baldwin J, Linton L, Lander ES (2000). An SNP map of the human genome generated by reduced representation shotgun sequencing. Nature.

[B10] Wiedmann RT, Smith TPL, Nonneman DJ (2008). SNP discovery in swine by reduced representation and high throughput pyrosequencing. BMC Genet.

[B11] Holt RA, Jones SJM (2008). The new paradigm of flow cell sequencing. Genome Res.

[B12] Warren RL, Sutton GG, Jones SJM, Holt RA (2007). Assembling millions of short DNA sequences using SSAKE. Bioinformatics.

[B13] Dohm JC, Lottaz C, Borodina T, Himmelbauer H (2007). SHARCGS, a fast and highly accurate short-read assembly algorithm for de novo genomic sequencing. Genome Res.

[B14] Hernandez D, François P, Farinelli L, Osterås M, Schrenzel J (2008). De novo bacterial genome sequencing: millions of very short reads assembled on a desktop computer. Genome Res.

[B15] Zerbino DR, Birney E (2008). Velvet: algorithms for de novo short read assembly using de Bruijn graphs. Genome Res.

[B16] Butler J, MacCallum I, Kleber M, Shlyakhter IA, Belmonte MK, Lander ES, Nusbaum C, Jaffe DB (2008). ALLPATHS: de novo assembly of whole-genome shotgun microreads. Genome Res.

[B17] Chaisson MJ, Pevzner PA (2008). Short read fragment assembly of bacterial genomes. Genome Res.

[B18] Farrer RA, Kemen E, Jones JDG, Studholme DJ (2009). De novo assembly of the Pseudomonas syringae pv. syringae B728a genome using Illumina/Solexa short sequence reads. FEMS Microbiol Lett.

[B19] Li H, Ruan J, Durbin R (2008). Mapping short DNA sequencing reads and calling variants using mapping quality scores. Genome Res.

[B20] Griffin DK, Robertson LB, Tempest HG, Vignal A, Fillon V, Crooijmans RPMA, Groenen MAM, Deryusheva S, Gaginskaya E, Carré W, Waddington D, Talbot R, Völker M, Masabanda JS, Burt DW (2008). Whole genome comparative studies between chicken and turkey and their implications for avian genome evolution. BMC Genomics.

[B21] Reed KM, Faile GM, Kreuth SB, Chaves LD, Sullivan LM (2008). Association and in silico assignment of sequences from turkey BACs. Anim Biotechnol.

[B22] Chaves LD, Knutson TP, Krueth SB, Reed KM (2006). Using the chicken genome sequence in the development and mapping of genetic markers in the turkey (Meleagris gallopavo). Anim Genet.

[B23] Illumina http://www.illumina.com/.

[B24] Smith AFA, Green P RepeatMasker. http://www.repeatmasker.org.

[B25] Schwartz S, Kent WJ, Smit A, Zhang Z, Baertsch R, Hardison RC, Haussler D, Miller W (2003). Human-mouse alignments with BLASTZ. Genome Res.

[B26] Applied Biosystems http://www.appliedbiosystems.com/.

[B27] Haubold B, Wiehe T (2006). How repetitive are genomes?. BMC Bioinformatics.

[B28] International Chicken Genome Sequencing Consortium (2004). Sequence and comparative analysis of the chicken genome provide unique perspectives on vertebrate evolution. Nature.

[B29] Illumina (2006). Protocol for Whole Genome Sequencing using Solexa Technology. BioTechniques Protocol Guide.

[B30] Zhang Z, Schwartz S, Wagner L, Miller W (2000). A greedy algorithm for aligning DNA sequences. J Comput Biol.

[B31] Nenfedov M, Zhu B, Thorsen J, Shu CL, Cao Q, Osoegawa K, Jong Pd (2003). New chicken, turkey, salmon, bovine, porcine and sheep genomic BAC libraries to complement world wide effort to map farm animals genomes. Plant and Animal Genome XI Conference Scherago International.

[B32] Green P Phrap. http://www.phrap.org.

