# Supplementary figures and images for: Large scale single nucleotide polymorphism discovery in unsequenced genomes using second generation high throughput sequencing technology: applied to turkey
Source: BMC Genomics. 2009 Oct 16;10:479. doi: 10.1186/1471-2164-10-479 (PMC2772860; doi:10.1186/1471-2164-10-479)

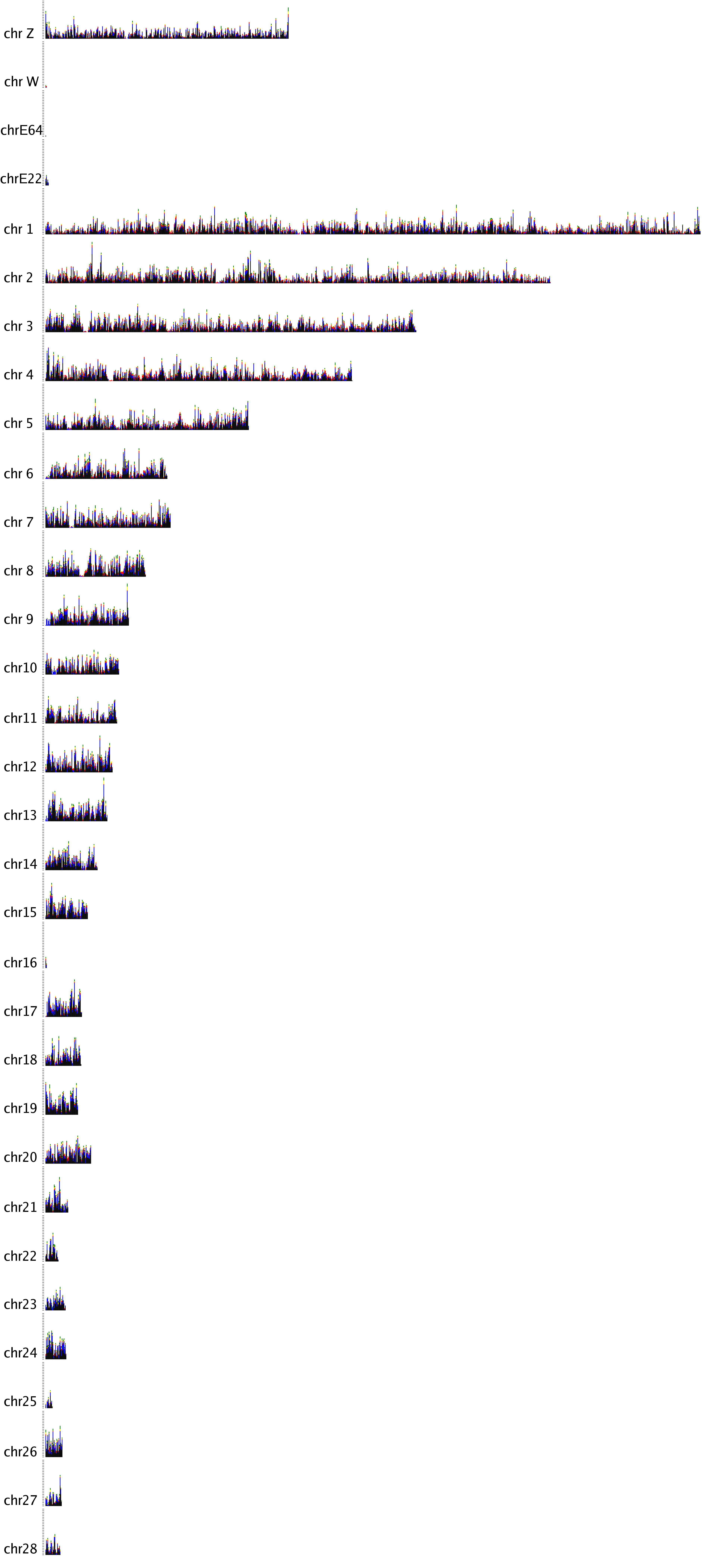

Supplement: Additional file 1 — Distribution of short read turkey contigs, turkey BES, and SNPs on chicken chromosomes. In black, short read contigs <100 bp; in blue, short read contigs ≥ 100 bp; in red, BES; in yellow, BES-short-read contigs; and in green, SNPs. On the X-axis, the chicken genome in 200 kb intervals. On the Y-axis, the frequency of mapped turkey features for a specific chicken genome interval. [file 1471-2164-10-479-S1.PNG]
