# Supplementary material for: Large scale single nucleotide polymorphism discovery in unsequenced genomes using second generation high throughput sequencing technology: applied to turkey
Source: BMC Genomics. 2009 Oct 16;10:479. doi: 10.1186/1471-2164-10-479 (PMC2772860; doi:10.1186/1471-2164-10-479)
Supplement: Additional file 2 — Primer sequences, PCR product sizes, and number of SNPs confirmed per amplicon for the 25 loci evaluated for genome similarity and SNPs. 1C = chicken, T = turkey. First, 13 loci were used in the determination of genome conservation between chicken and turkey. Next, 12 loci were used to validate the contig assembly and SNP detection procedure. [file 1471-2164-10-479-S2.DOC]

| **Locus (chicken genome)** | **species^1^** | **Forward, reverse primers, 5’to 3’** | **Amplicon size [bp]** | **Number of SNPs**  **scored** |
| --- | --- | --- | --- | --- |
| chr24-2166431-2167191 | C | ATTGCGTGCACTGAAAAATC, TCCTGGTCAATTGGAAATAGG | 641 | NA |
|  | T | ATTGCGTGCACTGAAAAATC, TCCTGGTCAATTGGAAATAGG | 641 | NA |
| chr5-52501937-52502445 | C | GAGCAGGGGAATTTGATGAG, CTCCCCTCATCTGCTTTCTG | 445 | NA |
|  | T | GAGCAGGGGAATTTGATGAG, CTCCCCTCATCTGCTTTCTG | 446 | NA |
| chr24-3088772-3089090 | C | AGGGTCAACGGCAACTAATG, CCCCGCTCTCCTCCCATTTC | 283 | NA |
|  | T | AGGGTCAACGGCAACTAATG, CCCTTCTCTCCTCCCATTTC | 283 | NA |
| chr6-27271758-27271981 | C | GCAGCGAGATGAAAGCTGAC, GAGAAAACACTGGGCAGCAG | 214 | NA |
|  | T | GCAGCGAGATGAAAGCTGAC, GAGAAAACATTGGGCAGCAG | 214 | NA |
| chr6-27271758-27271981 | C | CCAGCGCAGCGAGATG, GAGAAAACACTGGGCAGCAG | 237 | NA |
|  | T | CCAGCGCAGCGAGATG, GAGAAAACATTGGGCAGCAG | 237 | NA |
| chr1-111247460-111248400 | C | TGCAATTATTGATGCAATGG, AGGTGCCTCAGAGTGGAAAC | 730 | NA |
|  | T | TGCAATTATTGATGCAATGG, AGGTGCCTCAGAGTGGAAAC | 739 | NA |
| chr5-57714243-57714822 | C | GGAGCAGTAAGATGGCATGAG, ACAGAGCCTTTGGGATAAGG | 458 | NA |
|  | T | GGAGCAGTAAGATGGCATGAG, ACAGAGCCTTTGGGATAAGG | 457 | NA |
| chr12-1058107-1058700 | C | AAAACACCCTTGCCTCCAC, TTTCTGCTTCTCACGTCACC | 434 | NA |
|  | T | AAAACACCCTTGCCTCCAC, TTTCTGCTTCTCACGTCACC | 436 | NA |
| chr6-32485001-32485581 | C | TAAACGTGTCCATTTGGAAC, TGGATACCTGTAGCATTCTCTGC | 419 | NA |
|  | T | TAAACGTGGCCATTTGGAAC, TGGATACCTGTAGCATTCTCTGC | 416 | NA |
| chrZ-38116691-38117182 | C | AGAGCAGAGGCAATTTCACC, TCCTCATGAATAAAATGCCAAC | 408 | NA |
|  | T | AGAGCAGAGGCAATTTCACC, TCCTCATGAATAAAATGCCAAC | 410 | NA |
| chr4-597075-597843 | C | AACAATAGGACTGCCTCGAC, AAAGGCCAGACTATTACCTAGCAG | 744 | NA |
|  | T | AACGATAGGACTGCCTCGAC, AAAGGCCAGACTATTACCTAGCAG | 740 | NA |
| chr9-2591968-2592511 | C | TTTTCCAGATTTTCCAGATAGTCC, CCACCAACGAGTGAACTTTGTC | 453 | NA |
|  | T | TTTTCCAAATTTTCCAGATAGTCC, CCACCAACGAGTAAACTTTGTC | 453 | NA |
| chr1-37869034-37869342 | C | CTGATGGCAGAAGGGTTTG, TCATGTTCCTTGACTCTTCTTG | 257 | NA |
|  | T | CTGATGGCAGAAGGGTTTG, TCATGTTCCTTCACTCTCCTTG | 257 | NA |
| chr2-31180625-31180906 | T | CAAAGCAATGTGTCCGTAGG, GACCCACACATCCTTGATGG | 285 | 2 |
| chr5-57483295-57483518 | T | TGCAATGGAACTTGGAAACA, TGCTGGAGATGAACCGTGT | 224 | 2 |
| chr5-61952560-61952768 | T | TAGTTTAGGCTGCCCCACTG, GCAGCCATCTCTTCCCTCTT | 215 | 1 |
| chr3-13519590-13519965 | T | GCATCTTTTGGTCTGTTTTCTG, CAGTTGGATGATTTTAGCAACA | 377 | 4+1 |
| chr21 5257729-5258312 | T | GGGCTGAAATGCCATTGA, CTTGACCATTTCTTTTACCTGAA | 399 | 2 |
| chr6-17124476-17124765 | T | TCTCTGGTCCCCAGGAGAG, TTCAGGCTCATCTTTCAACAG | 336 | 3 |
| chr2-44384405-44384813 | T | TCCACAGTGTGAGGCTGTTC, AGCCTGGTCTGGTGGGTAAC | 461 | 3+1 |
| chr2-44103196-44103508 | T | CCCCCTAGCTAAATCCACAAC, ACAGAGCAGGAGCAGACCAC | 319 | 2+1 |
| chr6-12361082-12361463-565 | T | AGTCTGAGCTACAATTTCAGAGC, ATGTGTTTTTGCAACCCAGAG | 401 | 4 |
| chr5-45928735-45929043 | T | CTGATGTGCTTTATGCTGTGG, GCCTTCAGGGCTATCCAAAC | 308 | 1+1 |
| chr1-150077893-150078295 | T | GCAGTTCTTGGAAACTGTACG, CATGTGTGGGATTCTCTCCTC | 404 | 2 |
| chr26-3500373-3500846 | T | ATTCAGGTTGGCCTGAAGTG, TCAGCCAAAACAGTGTCACG | 505 | 3+1 |
